# Psychiatrical assessment of civil status of recipients of inpatient social services in two regions of Russia

**DOI:** 10.1192/j.eurpsy.2021.1882

**Published:** 2021-08-13

**Authors:** O. Rusakovskaya, A. Petrov, N. Korensky, N. Romashkina

**Affiliations:** 1 Forensic Psychiatry In Civil Process, V. Serbsky National Medical Research Centre for Psychiatry and Narcology, Moscow, Russian Federation; 2 Department Of Forensic Psychiatry, Krasnoyarsk Region Neuropsychiatric Facility, Krasnoyarsk, Russian Federation; 3 National Research Centre For Narcology, V. Serbsky National Medical Research Centre for Psychiatry and Narcology, Moscow, Russian Federation; 4 National Research Centre For Psychiatry, V. Serbsky National Medical Research Centre for Psychiatry and Narcology, Moscow, Russian Federation

**Keywords:** severe mental disorders, National Survey, institutional care, capacity

## Abstract

**Introduction:**

In 2019 national survey - personal examination of persons, living in residential facilities for mentally disabled people - was executed under the instruction of the Government by specialists of V.Serbsky National Medical Research Centre. For 2559 legally incapacitated residents the procedure of rehabilitation of full or diminished capacity was recommended. For 16132 legally capable residents the procedure for recognising their incapacity was recommended (Kekelidze ZI et al., 2020).

**Objectives:**

To compare the recommendations on legal capacity in two regions with different systems of inpatient social services.

**Methods:**

Full-sized noncontrol observational descriptive screening study.

**Results:**

In table 1 the recommendations on legal capacity in two regions are presented.

**Table:** 

Table 1. Recommendations on legal capacity		
	**Region 1**	**Region 2**
**Residents, were examined**	3837 (100%)	1859 (100%)
**Legally incapacitated residents**	3671 (95,7%)	1347 (72,5%)
**Partly legally capable residents**	17 (0,4%)	0 (0%)
**Legally capable residents**	149 (3,9%)	512 (27,5%)
**Rehabilitation of full or diminished capacity was recommended (% of incapacitated)**	186 (5%)	31 (2,3%)
**Deprivation of legal capacity was recommended (% of legally capable)**	12 (8%)	29 (5,7%)

**Conclusions:**

Differences in quantity of residents, for whom rehabilitation of full or diminished capacity was recommended, depend on characteristics of the systems of institutional care in the regions. In the first region a complex system of rehabilitation and deinstitutionalization has been organised. In the second region such system is just organising. Among legally capable and those, for whom rehabilitation of capacity was recommended, there were residents, able to live independently or under community-based services.

**Disclosure:**

No significant relationships.

